# Surgery

**Published:** 1910-03

**Authors:** C. Ferrier Walters


### SURGERY.

**The treatment of growths of the bladder,** particularly of those malignant in type, leaves much to be desired, and the by no means satisfactory results obtained are a sufficient plea for the more radical methods of treatment which for some years have been advocated and practised by an increasing number of continental and American surgeons well versed in genito-urinary work.

These procedures have made but little headway in this country, probably from the fact that the first attempts in the direction of more radical operations proved disastrous, producing an unwarrantable mortality, which with an improved technique, although still high, is gradually falling to a more reasonable figure, justifying the application of the more thorough methods of operation to the bladder, which has been applied to malignant growths elsewhere.

The most complete statistics on the results of operative treatment of bladder tumours are those compiled by Watson,[1] who collected the results of operative interference on 653 cases of vesical growths, with a view to substantiating his proposal to substitute bilateral lumbar nephrostomy and the establishment of renal fistulæ in cases of bladder tumours, for urethral implantation in connection with bladder resection or total extirpation, and that the bladder operation be done after an interval, and not together with the nephrostomies.

Of these cases 243 were benign and 410 malignant, the former class including papillomata, 203; myoma, 20; myxoma, 16; adenoma, 1; angioma, 2; the latter, carcinomata 358 and sarcomata 52.

He studied them with reference to the mortality attending each special operative method, in each special variety of tumour and the frequency of recurrence and freedom from recurrence that are seen in connection with the performance of each special operative method and each type of tumour.

He shows that the operative mortality in benign tumours apart from myoma is 12 per cent., with these 17 per cent., that in carcinoma it is 27 per cent., and in sarcoma 63 per cent.

With regard to the mortality, with reference to the type of operation and to that of the tumour, his statistics show that, in operation on so-called benign papillomata via the urethra, the mortality is 9 per cent., and in carcinoma 20 per cent.

With suprapubic operations in which resection was not performed the mortality in papillomata was 11.3 per cent., in myomata 15.3 per cent, in carcinomata 28 per cent., and in sarcoma 63 per cent.

By suprapubic methods, with partial resection of the bladder,

[1] Watson, *Ann. Surg.*, 1905, xlii, 806.

benign growths, including myoma, showed a 9.5 per cent. mortality, and carcinoma a mortality of 18.6 per cent.

Total extirpation of the bladder in twenty-five cases gave a mortality of 56 per cent.

Watson calls attention to the interesting fact which these statistics show in connection with the mortality of suprapubic operations, that in carcinoma, without resection, the death rate was 28 per cent., but with resection fell to 18.6 per cent ; in papilloma, without resection, 11.3 per cent., and with, but 9.5 per cent.

The lowest mortality shown is that of benign tumours removed by partial resection when operated on through the female urethra or the perineal urethra in the male.

He next takes the freedom from recurrence for more than one year according to the nature of the operation and the tumour, and finds the percentage in papillomata removed by urethral methods to be 28.2 per cent., by suprapubic operations, without resection, 27.5 per cent., and with resection 37.5 per cent.

In carcinomata removed suprapubically without resection freedom from recurrence for more than one year was 31.8 per cent., with resection 21 per cent. ; but with total cystectomy 54.5 per cent. remained free from recurrence for more than one year.

He sums up the results of operative treatment to the present time as follows :—

If the operative deaths and rapid recurrences are combined under the one heading of operative failures, such are seen to have occurred in 28.6 per cent. of benign tumours, exclusive of myoma, and in 46 per cent. of carcinomata.

He considers the question of the advisability of operating on these cases radically or not, and gives the following considerations that weigh for and against operative treatment, namely :—

That the condition if not removed is necessarily fatal, and beyond palliative treatment. That it is a suffering condition in the later stages, which in a number of cases palliative treatment cannot relieve. That there are a few patients who have been apparently wholly cured, and many more for whom radical operations have secured long intervals of freedom from suffering and symptoms. That the tendency to hydro- and pyo-nephrosis being frequently associated with bladder tumours, from involvment of the ureteric orifices, early operation would probably prevent these conditions. Also that tumours of the bladder are believed to change from a benign to a malignant type. Against these considerations for the operation he places the following : The large proportion of operative failures and the high mortality. The fact that in many cases of benign, and in some malignant tumours, there is a long interval between the appearance of the first symptoms and the time at which the patient's suffering is enough to cause him to seek advice. The futility of operating in cases of carcinoma in which metastases are already present, and the difficulty of determining whether they exist or not. Lastly, the hopelessness of operating in myxoma and sarcoma. He considers that the factors for operation outweigh those against it, provided that the growths dealt with are benign, or carcinoma which is apparently limited to the bladder, an opinion which is held by Albarran,[1] Young, etc.

As the percentage of recurrences is so large under the present operative methods, more radical treatment is indicated both in benign tumours, which after removal have recurred, and in malignant growths.

The mortality of total extirpation is very high, and apparently largely due to the methods of implantation of the ureters that have up to the present time been adopted, together with the fact that these have been carried out at the same time as the cystectomy, greatly increasing the shock of operation. Albarran[2] states that in the first twenty-six total cystectomies seven patients died from shock, which he considers due to a large extent to this means of prolonging the operation. Various sites have been used for implantation of the ureters.

Bardenheuer[2] in 1887, who was the first to perform total cystectomy, transplanted them into the abdominal wall, but the patient died of uræmia in fourteen days.

Pawlick[3] in 1888 successfully removed the whole bladder, implanting the ureters into the vagina; the patient was alive fourteen years later. The operative mortality is apparently influenced by the site of the implantation, and seeing that when in simple vesico-vaginal fistula the ureters are implanted into the rectum a mortality of 40 per cent. ensues, it is not surprising that it should reach the impossible figure of 76 per cent. following total cystectomy when implanted in that position.

Implantation into the vagina gives somewhat better results, the liability to sepsis and kinking of the ureters being less. Implantation into the urethra has been done on four occasions, with three deaths from shock.

When carried into the wound contraction is likely to occur at the orifice, and the diversion of the course of the ureters aids in producing hydronephrosis.

As an alternative to these methods Watson offers bilateral permanent nephrostomy as probably a more satisfactory proceeding than transplantation of the ureters, and for these reasons: That in Schmieden's statistics, quoted by Watson,[4] of 626 cases of nephrostomy, done for various conditions of the kidney, together with 353 he himself collected, the mortality was found to be but 15 per cent.

[1] Albarran, "Médecine Opératoire des Voies Urinaires," 1909.
[2] Quoted by Albarran, *Op. cit.*
[3] Pawlick, *Wien. med. Wchnschr.*, 1891, xli. 1,814.
[4] Watson, *Ann. Surg.*, 1905, xlii, 806.

As a result of these findings he advocates nephrostomy at intervals of a few weeks on each kidney, as a preliminary to extirpation of the bladder in suitable cases, which should be performed some four to six weeks later.

Apparently the best site for ligation of the ureters in nephrostomy is at a point as near the kidney pelvis as possible.

An excellent description of the technique of total cystectomy is given by Albarran in his "Médecine Opératoire des Voies Urinaires," 1909. Briefly it is as follows :—

The bladder is distended with fluid, the patient being in the position for perineal prostatectomy, a transverse incision is made in front of the anus, and through this the prostate and base of the bladder are separated from the rectum as high as possible. The Trendelenburg position is then adopted, and the bladder, through a transverse hypogastric incision, is separated from the peritoneum down to the perineal wound. By traction through the perineal wound the separation of the anterior part of the bladder is facilitated. The ureters and lateral vascular pedicles are then divided, and lastly the urethra cut across with the thermocautery.

The operation is more easily performed in the female, the first incision being through the anterior vaginal wall.

Pawlick, as cited by Albarran, proceeds in a similar manner, but implants the ureters into the anterior vaginal wall, and sutures the anterior and posterior walls of the vagina to the corresponding walls of the divided urethra, the vagina forming a receptacle for the urine.

Recent total cystectomies, adopting Watson's suggestion of preliminary nephrostomies, have been successfully performed by Cabot,[1] Young,[2] Genersich,[3] Rorsing.[4]

With regard to partial cystectomy, which Watson's statistics show without doubt to be indicated in benign tumours, both in respect to mortality and freedom from recurrence Albarran's[5] description is an excellent one, and briefly is as follows :—

The transverse incision is employed, the bladder opened, after having reflected the peritoneum beyond the growth, which is then, according to its position, removed from without in or *vice versa.* The first method is employed when the tumour is situated on the anterior or lateral wall. The latter when the growth lies at the neck or in the region of the ureteric orifices. When involving the ureteric orifice, this, with the growth, is removed, and the cut end of the ureter implanted into the site of the incision ; but when the ureter needs transplanting, and is not involved in the growth,

[1] Cabot, H., *Tr. Am. Ass. Genito-Urin. Surgeons*, 1909, iv, 125.
[2] Young, *Ibid.*, 1908, iii, 76.
[3] Genersich, "Urologia" (Budapest), 1907–8, 45–47.
[4] Rorsing, *Ztschr. f. Chir.*, 1907, xxxi, 94.
[5] *Op. Cit.*

it is secured into a separate button-hole made in the bladder wall away from the excision wound.

The intraperitoneal method of resection has recently sprung into vogue. In 1893 Harrington[1] recorded a successful intraperitoneal partial cystectomy. Mayo[2] in 1908 reported five cases of successful transperitoneal partial cystectomy, Scudder[3] four cases, and Primrose[4] and Cabot[5] single cases by this method. Van Ruediger Rydygier[6] has also practised this method. Berg[7] advocates and has successfully practised this method, but in addition, as a preliminary, removes all the pelvic glands and lymphaties likely to be infected.

It will, perhaps, not be out of place here to offer a suggestion, which I have been able to carry out practically in one case with some success, for the treatment of inoperable cases of the infiltration type of carcinoma of the bladder, in which the condition is almost unbearable from the associated cystitis giving rise to intolerable pain, occurring every few minutes with the frequency of micturition that usually exists. It has been observed in such cases that when the growth extends to the neck of the bladder incontinence ensues, and with the establishment of this condition the agonising pain of spasm of the neck of the bladder disappears. This suggested to me that permanent dilatation of the urethra might relieve this almost unbearable condition, so I proceeded to widely dilate the urethra in a woman thus afflicted, with the result that all the pain produced by the frequent micturition ceased, and she slept throughout the night, which she had been unable to do for months, thus apparently justifying one in such a procedure, for the suffering is sometimes so great in these cases that morphia fails to relieve it, and Fenwick[8] has suggested that even division of the spinal cord in the lumbar region is justifiable as a means of relieving it.

C. FERRIER WALTERS.

[1] Harrington, *Ann. Surg.*, 1893, xviii, 408.
[2] Mayo, *Ibid.*, 1908, xlviii, 105.
[3] Scudder, *Ibid.*, 1908, xlviii, 862. [4] Primrose, *Ibid.*, 1909, l, 1137.
[5] Cabot, A. T., *Tr. Am. Ass. Genito-Urin. Surgeons*, 1909, iv, 119.
[6] Rydygier, *Zentrabl. f. Chir.*, 1909, xxxvi, 372.
[7] Berg, *Ann. Surg.*, 1904, xl, 382.
[8] Fenwick, "Handbook of Clinical Cystoscopy," 1904.

